# Simultaneous Adsorption of Cationic Dyes from Binary Solutions by Thiourea-Modified Poly(acrylonitrile-*co*-acrylic acid): Detailed Isotherm and Kinetic Studies

**DOI:** 10.3390/ma12182903

**Published:** 2019-09-08

**Authors:** Abel Adekanmi Adeyi, Siti Nurul Ain Md Jamil, Luqman Chuah Abdullah, Thomas Shean Yaw Choong, Kia Li Lau, Mohammad Abdullah

**Affiliations:** 1 Department of Chemical and Environmental Engineering, Faculty of Engineering, Universiti Putra Malaysia, UPM Serdang 43400, Malaysia; abeladeyi@abuad.edu.ng (A.A.A.); chuah@upm.edu.my (L.C.A.); csthomas@upm.edu.my (T.S.Y.C.); laukiali@hotmail.com (K.L.L.); 2 Department of Chemical and Petroleum Engineering, College of Engineering, Afe Babalola University Ado-Ekiti, ABUAD, KM. 8.5, Afe Babalola Way, PMB 5454, Ado-Ekiti, Ekiti State 360211, Nigeria; 3 Department of Chemistry, Faculty of Science, Universiti Putra Malaysia, UPM Serdang 43400, Malaysia; 4 Centre of Foundation Studies for Agricultural Science, Universiti Putra Malaysia, UPM Serdang 43400, Malaysia; 5 Institute of Tropical Forestry and Forest Products (INTROP), Universiti Putra Malaysia, UPM Serdang 43400, Malaysia; 6 Faculty of Chemical Engineering, Universiti Teknologi Mara, 81750 Masai, Johor Darul Takzim, Malaysia; moham3767@johor.uitm.edu.my

**Keywords:** adsorption, poly(acrylonitrile-*co*-acrylic acid), thiourea, cationic dyes, binary system, isotherms, kinetics

## Abstract

In this study, simultaneous adsorption of cationic dyes was investigated by using binary component solutions. Thiourea-modified poly(acrylonitrile-*co*-acrylic acid) (TMPAA) polymer was used as an adsorbent for uptake of cationic dyes (malachite green, MG and methylene blue, MB) from aqueous solution in a binary system. Adsorption tests revealed that TMPAA presented high adsorption of MG and MB at higher pH and higher dye concentrations. It suggested that there are strong electrostatic attractions between the surface functional groups of the adsorbent and cationic dyes. The equilibrium analyses explain that both extended Langmuir and extended models are suitable for the description of adsorption data in the binary system. An antagonistic effect was found, probably due to triangular (MG) and linear (MB) molecular structures that mutually hinder the adsorption of both dyes on TMPAA. Besides, the kinetic studies for sorption of MG and MB dyes onto adsorbent were better represented by a pseudo-second-order model, which demonstrates chemisorption between the polymeric TMPAA adsorbent and dye molecules. According to experimental findings, TMPAA is an attractive adsorbent for treatment of wastewater containing multiple cationic dyes.

## 1. Introduction

Rapid growth in population and industrial activities have ensued in accumulation of pollutants in the environment due to their waste disposal without any treatment [1,2]. It is reported that about 1.2 trillion tons of non-treated industrial wastewater and storm water are discharged into the environment per annum [3,4]. Industries such as paint, textile, paper, rubber, leather, petroleum, pharmaceutical, and food are major contributors, enhancing the concentration of contaminant present in wastewater [5,6,7,8]. Over 100,000 synthetic dyes—cationic, anionic and dispersive—have been broadly used in the aforementioned industries generating volumes of wastewater annually. They are by design highly stable molecules not easy to biodegrade, made to resist degradation by chemical, biological, and light exposure, as well as their multifarious chemical structure [9]. These pose a big challenge and are harmful to ecological and environmental systems-reducing light penetration and photosynthesis [10,11]. Under anaerobic conditions, some cationic dye-bearing wastewater breaks down into hazardous aromatic amines, causing critical health problems to animals and humans [12]. Treatment of industrial wastewater especially dye bearing wastewater in order to meet stringent discharge regulations in industrial operations is a major concern globally.

Various developed technologies are available for isolation of dye ions present in wastewater such as anaerobic decomposition, membrane separation, precipitation, electrocoagulation, and flocculation. Photo-oxidation and biological degradation has also been proposed and cited for the treatment of color containing effluents. Conversely, this method is relatively expensive and complex. Alternatively, adsorption remains the most common and efficient decontamination technology for dye bearing industrial effluents [13,14,15]. Many researches have been made on the possibility of adsorbents using mineral sorbents, activated carbon, peat, chitin, rice husk, soy meal hull, and other agricultural wastes. The sorption capacity of these adsorbents is not very effective to improve adsorption performance [16]. Preparation of new polymer-based adsorbents with functional groups that have significant effects on the efficiency, selectivity, and reusability is of utmost concern for researchers, due to the germane role of the adsorbent in adsorption technique. Several chelating resins have been prepared through grafting of monomers onto natural or synthetic polymer matrix and conventional polymerization of the monomers such as acrylonitrile (AN), acrylic acid (AA), methacrylic acid (MA), and divinylbenzene (DVB-80) [17]. Furthermore, chemical modification of a synthesized polymer milieu by a chelating moiety such as amine, thiourea, and amino-acids has also been used to produce new/modified polymeric adsorbents [15,16,18,19]. In such studies, the carboxyl and amine groups have been established to be two most effective functional groups for removal of organic and inorganic pollutants from aqueous solutions.

Moreover, previous researches on dye adsorption were carried out explicitly in single dye systems and scanty study reports were available in binary dye systems. Our previous work reports in detail preparation and characterization of thiourea-modified poly(acrylonitrile-*co*-acrylic acid) (TMPAA) and its adsorption capacities towards MG and MB in a single dye system [20,21,22]. Generally, multiple components of dyes coexist in real industrial wastewater systems. This multiplicity in actual applications may affect the dye adsorption process either in antagonistic or synergistic manner [23,24]. Therefore, it is imperative to investigate adsorption of dye both in single and multiple dye systems. Herein, this study aims to assess TMPAA applicability for the adsorptive removal of cationic dyes from aqueous solution in binary systems. Binary adsorption isotherms and kinetics of MG and MB uptake by TMPAA polymer were also investigated.

## 2. Materials and Methods 

### 2.1. Chemicals and Reagents

**Figure 1:**
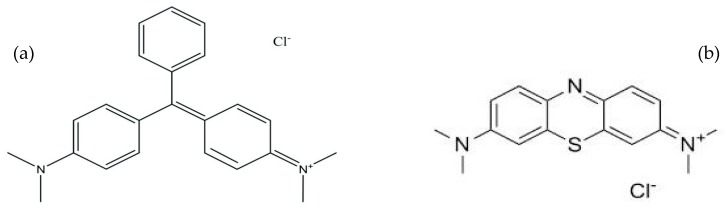
Molecular structures of (**a**) malachite green and (**b**) methylene blue.

All chemical reagents purchased were analytical grade, used without further purification except acrylonitrile (AN) and acrylic acid (AA) (Acros Organics, Morris, NJ, USA) purified by aluminium oxide (Merck, Darmstadt, Germany) with glass wool. Potassium persulphate (KPS) and sodium bisulphate (SBS) (R&M Chemicals, Essex, UK) were used as initiators for free radical polymerization. Methanol and ethanol were purchased from Systerm ChemAR (Shah Alam, Malaysia). Thiourea (R&M Chemicals, Essex, UK) was used to modify the synthesized copolymer. Hydrochloric acid and sodium hydroxide (R&M Chemicals, Essex, UK) were used for the pH adjustment. The two cationic dyes, malachite green (MG) and methylene blue (MB) purchased from Acros Organics, NJ, USA, were used as adsorbate. Their general properties and structures are shown in Table 1 and Figure 1, respectively.

### 2.2. Preparation of Thiourea-Modified Poly(acrylonitrile-co-acrylic acid)(TMPAA)

Poly(acrylonitrile-*co*-acrylic acid) was synthesized and modified according to previous work [21]. The feed ratio of monomers AN:AA was 97:3 (vol/vol). The reaction medium, 200 mL deionized water was initially purged with N_2_ gas for 30 min at 40 °C. Then, AN and AA were introduced into the reaction medium followed by KPS and SBS (as initiator). The solution was stirred mechanically at 200 rpm, and the reaction was allowed for 120 min. The produced copolymer (poly(AN-*co*-AA)) was soaked in methanol, filtered, and washed successively with methanol and deionized water. The copolymer was oven dried at 45 °C until a constant mass white powder.

For surface functionalization, 6.0 g of thiourea and ethanol/deionized water (1:2% volume) were mixed and stirred continuously at 200 rpm for 0.5 h at 70 °C. Then, 5.0 g of poly(AN-*co*-AA) was added to the solution for 5 h at 100 °C. Then, the resulting solids, thiourea-modified poly(AN-*co*-AA) (TMPAA), rinsed liberally in ethanol/deionized water solution, filtered, and dried at 50 °C to constant mass.

### 2.3. Binary Adsorption Studies 

Adsorptive tests of MG and MB cationic dyes on TMPAA in binary systems were performed in a batch mode. 100 mL solutions of dyes (50 mL of MG and 50 mL of MB) were prepared with one dye at a fixed concentration and the other at varying concentrations. One set of tests were performed at fixed MB concentration of 20 mg/L and second set at 100 mg/L of MB, while various MG concentrations at 20–100 mg/L. Under constant stirring speed of 100 rpm, the solutions were controlled at 25 °C, and 100 rpm agitation speed. The other set of experiments was carried out at constant MG concentrations (20 and 100 mg/L) while MB was varied from 20 to 100 mg/L. TMPAA at 0.5 g was mixed with the dye solutions and kept at 100 rpm, 25 °C. The solutions were withdrawn at several time intervals, filtered for dye concentration measurement. The absorbance was measured at two wavelengths of 617 and 665 nm by a Lambda 35 UV-Vis spectrophotometer. The following equations were used to determine the dye concentrations [25,26].

(1) $$C_{MG}=\frac{\left(K_{MB2}A_{1})-(K_{MB1}A_{2}\right)}{\left(K_{MG1}K_{MB2})-(K_{MG2}K_{MB1}\right)}$$

(2) $$C_{MB}=\frac{\left(K_{MG1}A_{2})-(K_{MG2}A_{1}\right)}{\left(K_{MG1}K_{MB2})-(K_{MG2}K_{MB1}\right)}$$

Where $K_{MG1}$ and $K_{MB1}$ represent the calibration constants for dyes MG and MB at $\lambda_{1,\max}$ while $K_{MG2}$ and $K_{MB2}$ represent the calibration constants for dyes MG and MB at $\lambda_{2,\max}$. $A_{1}$ and $A_{2}$ are the absorbance at wavelength $\lambda_{1,\max}$ and $\lambda_{2,\max}$, respectively.

The calculation of the extent of dye uptake, R(%), and adsorbent sorption capacity at equilibrium ($q_{e}$) condition can be expressed as:

(3) $$\%R=\frac{C_{o}-C_{e}}{C_{o}}\times 100$$

(4) $$q_{e}=\;V\frac{C_{o}-C_{e}}{m_{ads}}$$

*C_o_* (mg/L) and *C_e_* (mg/L) are the concentrations of the cationic dye at initial and equilibrium respectively; $q_{e}$ (mg/g) is the amount of dye adsorbed, V (L) is the volume of the dye solution and $m_{ads}$ (g) the weight of the TMPAA adsorbent used. All the experimental tests were performed thrice and the average value recorded.

### 2.4. Calculation of Adsorption Isotherms

The single adsorption isotherms were calculated using the Langmuir and Freundlich models. The Langmuir isotherm and Freundlich isotherm equations can be expressed linearly and respectively as [27,28,29]:

(5) $$\frac{C_{e}}{q_{e}}=\;\frac{1}{K_{L}\;q_{\max}}\;+\;\frac{C_{e}}{q_{\max}}$$

(6) $$\ln\;(q_{e})=\;\ln\;(K_{F})\;+\;\frac{1}{n}\;\ln\;(C_{e})$$

$q_{\max}$ (mg/g) is the Langmuir maximum adsorption capacity. $K_{L}$ and $K_{F}$ represent respectively Langmuir and Freundlich constants, and the heterogeneity factor is denoted by n.

The single component isotherm equations are extended and/or modified to investigate the interactions between the adsorption capacity of a component and the concentration of other components present in wastewater. The extended Langmuir model (also known as non-modified competitive Langmuir isotherm) for binary systems can be written as [30]

(7) $$q_{e,1}=\;\frac{q_{\max,1\;}K_{L,1}\;C_{e,1}}{1\;+\;K_{L,1}\;C_{e.1}+\;K_{L,2}\;C_{e,2}}$$

(8) $$q_{e,2}=\;\frac{q_{\max,2\;}K_{L,2}\;C_{e,2}}{1\;+\;K_{L,1}\;C_{e.1}+\;K_{L,2}\;C_{e,2}}$$

The parameters $q_{\max,1}$, $K_{L,1}$, and $K_{L,2}$ were evaluated for a series of experimental values of $q_{e,1}$ and $C_{e,1}$ by minimizing the error in non-linear regression analysis or applying solver function of non-linear regression in Microsoft Excel [31].

The extended Freundlich model is applied to multilayer heterogeneous adsorption systems when the interaction is occurring among the adsorbed components [31,32]. The extended Freundlich equation for a binary system is expressed as

(9) qe,1= KF,1 Ce,1(1n1) + x1Ce,1x1+ y1 Ce,2z1

(10) qe,2= KF,2 Ce,2(1n2) + x2Ce,2x2+ y2 Ce,1z2

$q_{e,1}$ and $q_{e,2}$ are the equilibrium adsorption capacity for solute 1 and 2 (mg/g), respectively. $C_{e,1}$ and $C_{e,2}$ represent the equilibrium concentration of component 1 and 2 (mg/L), respectively. The values of adsorption intensities $n_{1}$, $n_{2}$ and Freundlich constant $K_{F,1}$ and $K_{F,2}$ are obtained from the experimental data of individual Freundlich models. The values of constants $x_{1}$, $y_{1}$, $z_{1}$ and $x_{2}$, $y_{2}$, $z_{2}$ were obtained by minimizing error in non-linear regression analysis for a series of experimental values of $q_{e,1}$ versus $C_{e,1}$ and $q_{e,2}$ versus $C_{e,2}$ respectively [30,33].

### 2.5. Competition and Interaction Mechanism

Three distinct forms of interactions are feasible between the adsorbate molecules in a multicomponent adsorption system. Interaction effects are described based on the ratio of adsorption capacity of an adsorbent ($Q_{m}$) in a multicomponent solution to the sorption capacity of contaminant ($Q_{i}$) in a single component solution. The possible interactions among the adsorbate molecules are:

(a) *Antagonistic interaction*: This occurred when the adsorption capacity of an adsorbent reduces in a solution containing other components ($Q_{m}/Q_{i}$ < 1).
(b) *Synergistic interaction*: The adsorption capacity of an adsorbent increases when it is in association with other components ($Q_{m}/Q_{i}$ > 1).
(c) *Non-interaction*: The adsorption capacity is independent of the absence or presence of other components in a solution ($Q_{m}/Q_{i}$ = 1) [34,35].

Several principles and methods are employed to explain the interaction between the species in a binary adsorption system. These terms further describe how a component inhibits the adsorption of other components in the multi-component solution.

*P-Factor:* The P-factor model is a correlative technique developed by McKay and Al Duri (1987) and used to compare the monocomponent isotherm data with the binary isotherm data. The P-factor ($P_{fi}$) elucidates how the adsorption of a component is inhibited by other components in a binary mixture. It is given as:

(11) $$P_{fi}=\;\frac{Q_{i,s}}{Q_{i,b}}$$

$Q_{i,s}$ and $Q_{i,b}$ represent the sorbent capacity for the component i in the single component solution and the binary system. $P_{fi}$ value defined the nature of the interaction (inhibition, enhancement, or non-interference) between the two components. The value of $P_{fi}$ = 1 signifies unhindered interaction, $P_{fi}$ > 1 demonstrates that the adsorption of component i is inhibited in the presence of other solutes, while adsorption of component i enhanced at $P_{fi}$ < 1 [36,37,38].

*Inhibitory Effect***:** In a multicomponent adsorption system, entrapment of an adsorbate may be influenced by another and is delineated by inhibitory effect (ΔIE) given as

(12) $$\Delta IE=\;\frac{Q_{i,s}\;-\;Q_{i,b}}{Q_{i,s}}$$

The greater value of ΔIE indicates the suppression level of adsorption of one solute in the presence of another [37,39,40].

*Selectivity Ratio:* The affinity of an adsorbent towards a particular component in a binary system is indicated by selectivity ratio ($S_{\left(i/j\right)}$). Based on the morphology, surface structure, and pore distribution of an adsorbent, selectivity ratio investigates the adsorbent preference towards one solute in the presence of another [40,41,42]. The selectivity ratio is defined as

(13) $$S_{\left(i/j\right)}=\;\frac{Q_{i,b}}{Q_{j,b}}\;=\frac{Q_{i,s}}{Q_{j,s}}$$

Where $Q_{i,b}$ and $Q_{i,s}$ represent the adsorption capacity of the component i in the binary and single component solution. The value of $S_{\left(i/j\right)}$ being less than one implies that the adsorbent has more affinity towards component j than the component i [37].

### 2.6. Calculation of Adsorption Kinetics

In order to comprehend the nature and process of adsorption, pseudo-first-order and pseudo-second-order kinetic models were employed. The integral linear form of the Lagergren pseudo-first-order and Ho pseudo-second-order models can be expressed as: [43,44]

(14) $$\ln\;(q_{e}\;-\;q_{t})\;=\;\ln\;(q_{e})\;-\;k_{1}\;t$$

(15) $$\frac{t}{q_{t}}\;=\;\frac{1}{k_{2}\;q_{e}^{2}}\;+\;\frac{1}{q_{e}}\;t$$

$q_{e}$(mg/g), $q_{t}$(mg/g) are respectively represent adsorption capacity at equilibrium and at time t. $k_{1}$ (min.^-1^), and $k_{2}$(g/mg min.) are the rate constants for pseudo first-order and pseudo-second-order, respectively.

## 3. Results and Discussions

### 3.1. Characterization of TMPAA

Detail of the physical and chemical properties of TMPAA has been reported previously [21]. Presence of functional groups, OH and NH_2_ (3345 cm^−1^), -C-H (2935 cm^-1^), -C=O (1728 cm^−1^), and -C=S (729 cm^−1^), was revealed by FTIR spectrum. TMPAA was spherical with rough surface area as observed by SEM image. The adsorption isotherm of N_2_/77 K of the modified polymer corresponds to type IV and displayed the existence of narrow hysteresis loop. Mesoporous features of the TMPAA were confirmed by its BET surface area and pore size of 26.31 m^2^/g and 47.93 nm, respectively. The surface charge measurement was negative in both acidic and alkaline media.

### 3.2. Effect of Initial Dye Concentration and pH 

**Figure 2:**
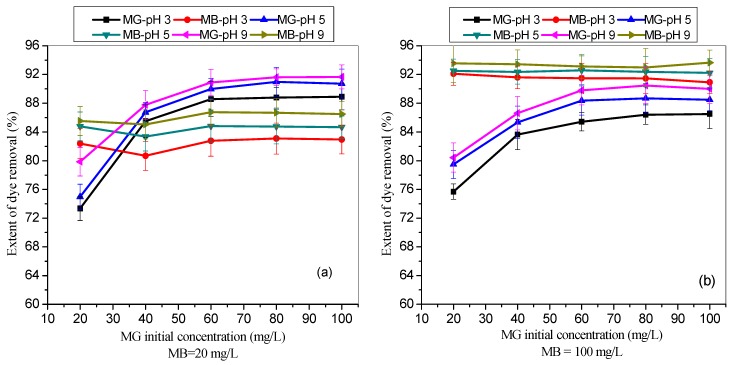
Effect of initial MG dye concentrations on the extent (%) of dye uptake by TMPAA at different pH in the presence of (**a**) 20 mg/L, and (**b**) 100 mg/L of MB (dose: 0.5 g/100 mL; agitation speed: 100 rpm; time: 2 hr.; temperature: 25 °C).

Figure 2 shows the influence of different initial MG dye concentrations on the extent (%) of removal of MG by TMPAA while keeping the concentration of MB at 20 mg/L (Figure 2a) and 100 mg/L (Figure 2b), respectively. Simultaneously, the effect of the solution pH was investigated by adjusting the adsorption systems to pH 3, 5, and 9, respectively. The MG uptake was increased from 73.36% to 88.92% (pH 3), 74.98% to 90.74% (pH 5) and 79.86% to 91.66% as the initial MG concentration was increased from 20 mg/L to 100 mg/L in the presence of 20 mg/L MB dye. At higher MB concentration (100 mg/L), the MG uptake was raised from 75.69% to 86.49% (pH 3), while 80.43%–89.99% removal increment was observed at pH 9.

As seen in Figure 2, the extent of MG dye uptake in a binary cationic dye solution improved with increasing concentration. It is due to enhancement in driving potency (induced by concentration gradient) required to subdue the resistance related to mass transfer between adsorbate and TMPAA adsorbent. Zhou and co-worker (2019) also report similar result trend for adsorption of rhodamine B (RhB), neutral red (NR), and MB cationic dyes onto sulfonated poly(arylene ether nitrile) (SPEN) based adsorbents [45]. Moreover, Figure 1 depicts that the extent of MB uptake was almost constant although the initial MG concentrations were increased from 20 mg/L.

**Figure 3:**
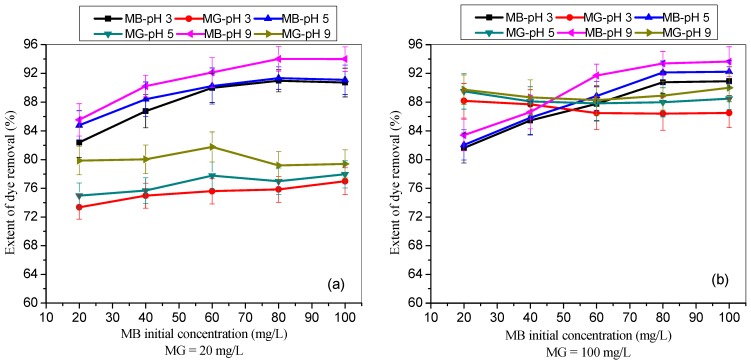
Effect of initial MB dye concentrations on the extent (%) of dye uptake by TMPAA at different pH in the presence of (**a**) 20 mg/L and (**b**) 100 mg/L of MG (dose: 0.5 g/100 mL; agitation speed: 100 rpm; time: 2 hr.; temperature: 25 °C).

Figure 3 shows the impact of different initial MB dye concentrations on the extent (%) MB uptake by TMPAA while keeping the concentration of MG at 20 mg/L (Figure 3a) and 100 mg/L (Figure 3b), respectively. The pH of the binary mixture was adjusted to 3, 5, and 9 to study the effect of pH on the extent of dye uptake by TMPAA adsorbent. The percentage MB removal (Figure 3a) was increased from 82.39% to 90.74% (pH 3), 84.78% to 91.10% (pH 5) and 85.52% to 93.99% (pH 9) as the initial MB concentration were raised from 20 mg/L to 100 mg/L in the presence of 20 mg/L MG dye. At higher MG concentration (100 mg/L, Figure 3b), the MB uptake was raised from 81.62% to 90.92% (pH 3), 82.04% to 92.23% (pH 5) while 83.41% to 93.65% uptake increment was recorded at pH 9.

Remarkably, the extent of MG and MB dye removal in the binary system increased with increasing solution pH (Figure 2 and Figure 3). This result is attributed to ionization of cationic dyes and TMPAA surface charge deprotonation [46]. The presence of thioamide groups on the polymeric adsorbent surface fully deprotonated in alkaline medium and negative charges was prompted in the polymer linkage. This negatively charged surface facilitates high sorption of positively charged cationic dyes from liquid phase due to an enhanced electrostatic pull. The low extent of dye uptake was observed in acidic condition due to protonation and increased the hydrophobicity of TMPAA microsphere. There was presence of additional positive charge at low pH, which reduced the uptake of cationic MG and MB dyes due to repulsive force coupled with TMPAA preferential adsorption of excess H^+^ ion compared to dye due to the smaller size. Similar adsorptive behaviour was reported by [47], indicating that adsorption of cationic dyes by poly(*N*-isopropylmethacrylamide-acrylic acid) microgels increased as the solution pH increases.

**Figure 4:**
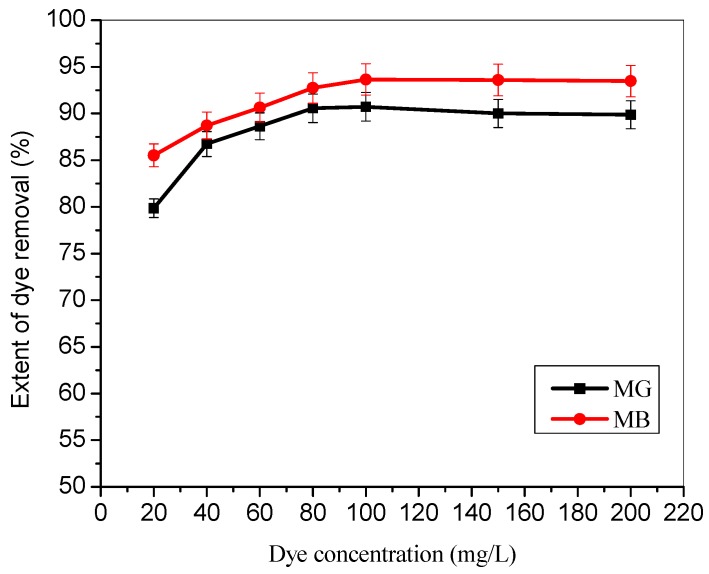
Effect of equal initial MG and MB dyes concentration on the extent of removal in a binary system (dose: 0.5 g/100 mL; agitation speed: 100 rpm; time: 2 hr.; temperature: 25 °C).

Generally, TMPAA adsorbents exhibit slightly higher adsorption preference towards MB dye compared to MG dye in a binary system (Figure 4), probably due to their triangular (MG) and linear (MB) molecular structures. The adsorption capacity of TMPAA towards both dyes increased as the initial concentration increases. This is due to higher driving force propelled by a concentration gradient that overcomes mass transfer resistance between the adsorbent surface and dye solution.

### 3.3. Effect of TMPAA Dose

**Figure 5:**
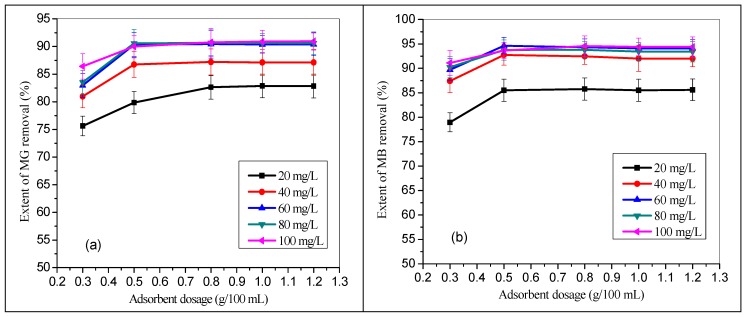
Effect of TMPAA dosage on the extent of dye uptake in a binary system (**a**) MG and (**b**) MB dyes (MG conc.= MB conc.; agitation speed: 100 rpm; time: 2 hr.; temperature: 25 °C).

The mass of adsorbent is a key parameter with a vital role in the adsorption process. To determine the optimum dosage of TMPAA, the polymeric adsorbent dose was varied from 0.3 g to 1.2 g/100 mL of binary MG and MB mixture, continuously stirred for 1 hr at room temperature. Figure 5a,b shows the extent of MG and MB in a binary system. The extent of dye uptake in both cases was first increased rapidly up to 0.5 g TMPAA dose, then became almost constant. This sudden increment was due to an increase in adsorptive surface area as well as the availability of more binding sites for MG and MB dyes adsorption [47]. The percentage of MG and MB uptake increased as initial concentration increases (Figure 5). Further increment in the concentration of TMPAA particles portrayed little or non-significant increase in the cationic dye entrapment. This phenomenon may be associated with the availability of less dye adsorbate to be adsorbed compared to excess available active binding sites of TMPAA.

### 3.4. Effect of Contact Time 

**Figure 6:**
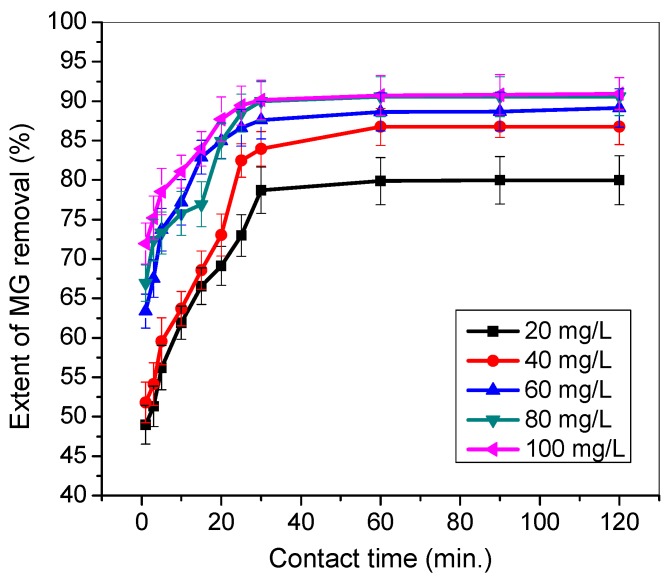
Effect of contact time on the adsorption of MG dyes in the presence of MB (temperature: 25 °C; agitation speed: 100 rpm; dosage: 0.5 g/100 mL; Conc. MG equal Conc. MB).

**Figure 7:**
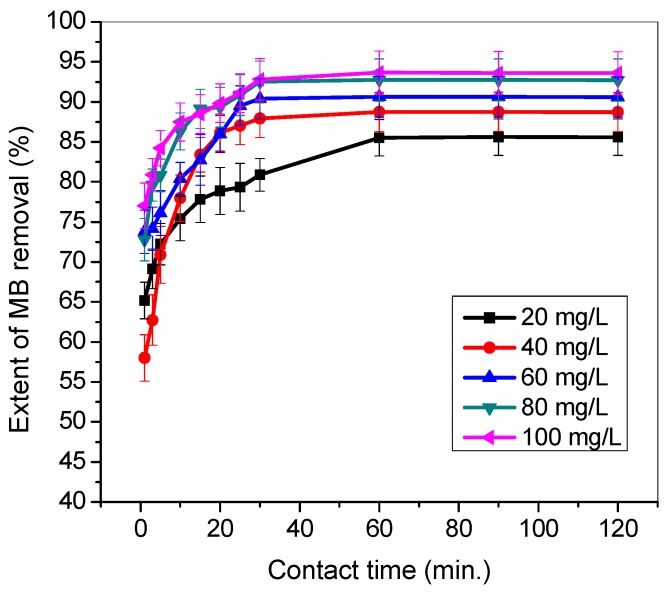
Effect of contact time on the adsorption of MB dyes in the presence of MG (temperature: 25 °C; agitation speed: 100 rpm; dosage: 0.5 g/100 mL; Conc. MG equal Conc. MB).

Determination of optimum contact or agitation time was performed at the varied initial concentration (20–100 mg/L) in a binary system. The results of optimum contact time for MG and MB uptake using TMPAA are presented in Figure 6 and Figure 7, respectively. The influence of residence time (0–120 min) on the extent of cationic dye uptake by TMPAA was investigated at pH 9, 0.5 g TMPAA dose and at 25 °C.

Initially, the removal percentage of MG was rapid in the first 30 min and then attained equilibrium after 60 min of agitation time. Equilibrium in the adsorption process achieved after 1 h due to saturation of active binding sites. A similar phenomenon was observed in the case of MB uptake in the presence of MG dye. Increase in MG and MB concentration resulted in a rise in the quantity of dyes entrapped. This is attributed to the fact that adsorption was directly proportional to more concentration gradient at the initial stage, where migration and convection led to greater mass transfer from the bulk solution to TMPAA surface and its reactive binding sites. This result trend is in agreement with the findings of Mishra et al. (2017); Alqadami et al. (2018); Asfaram et al. (2017) and Idan et al., (2017) who also worked on adsorption of dyes [48,49,50,51].

**Figure 8:**
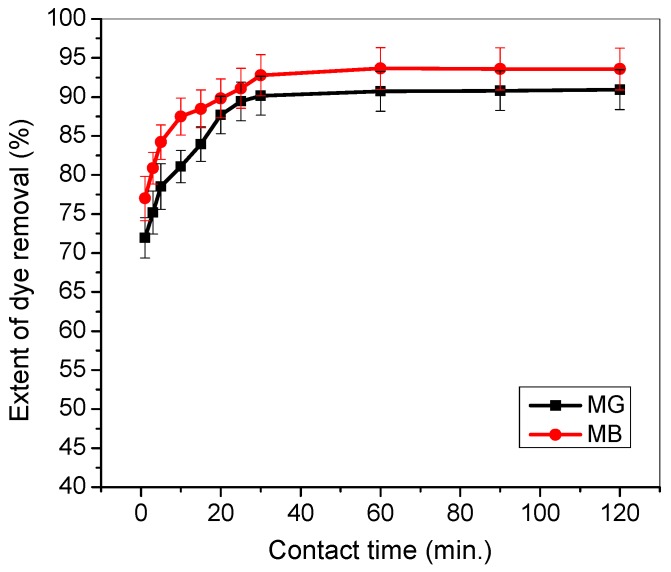
Effect of contact time on the adsorption of MG and MB dyes in a binary system (temperature: 25 °C; agitation speed: 100 rpm; dosage: 0.5 g/100 mL, Conc. MG equal Conc. MB: 100 mg/L).

The extent of dye uptake as a function of time for the two cationic dye was compared and illustrated in Figure 8 in a binary system. TMPAA demonstrates higher preference towards adsorption of MB than MG. Then both dyes attained equilibrium almost at the same residence time.

### 3.5. Adsorption Isotherms for Single and Binary Systems

Adsorption isotherm is fundamental to the design of adsorption systems; it demonstrates the relationship between a specific adsorbate and an adsorbent. The Langmuir and Freundlich isotherms parameters were calculated for a single adsorption system and presented in Table 2. Equilibrium data for a single adsorption study was well described by the Freundlich model.

Table 3 presents the equilibrium adsorption Langmuir model parameters for MG and MB dyes uptake by TMPAA in binary adsorption scheme. As expected, the maximum adsorption capacities, $q_{\max}$ of TMPAA for MG and MB dyes entrapment in binary solutions, were respectively found to be 150.97 mg/g and 124.61 mg/g. While 429.18 mg/g and 308.64 mg/g were the maximum adsorption capacities of TMPAA for MG and MB dyes, respectively in a single solution (Table 2). The results exhibit that the adsorption capacity of the modified polymer for cationic dyes declined in the binary system related to a single solution. This is attributed to the fact that in the binary system, partial or total competitions between adsorbate ions for the binding sites on TMPAA surface occur and act as the entrapment-governing aspect. Besides, adsorption affinity of the modified polymer surface is mutually hindered by lateral interaction and rivalry between MG/MB components for the occupancy of sorption site [31,52]. A similar observation was reported Kurniawan et al. (2012) for the uptake of basic dyes in a binary system by rarasaponin-bentonite [53]. Ziane and coworkers (2018) report reduction in adsorption capacities of modified dolomite (D900), for the removal of Reactive Black 5 (RB5) and Congo Red (CR) from wastewater containing a mixture of two dyes. According to their report, the single and binary maximum sorption capacities were 51.81 mg/g and 44.57 mg/g (RB5), and 261.36 mg/g and 153.04 mg/g (CR), respectively [54]. Also, the batch adsorption experiment performed by Maleki and team showed that the maximum adsorption capacity of amine-functionalized multi-walled carbon nanotubes for the uptake of Acid Blue 45 (AB45) and Acid Black 1 (AB1) dyes in binary system were 625 mg/g and 666 mg/g respectively, while the maximum adsorption capacity in single solution was 666 mg/g and 714 mg/g [15].

Isotherm studies of binary cationic MG and MB dyes adsorption were further analyzed using extended Langmuir equation (ELE) and extended Freundlich equation (EFE), respectively.

#### 3.5.1. Extended Freundlich Equation (EFE) for Binary Cationic Dye System

**Figure 9:**
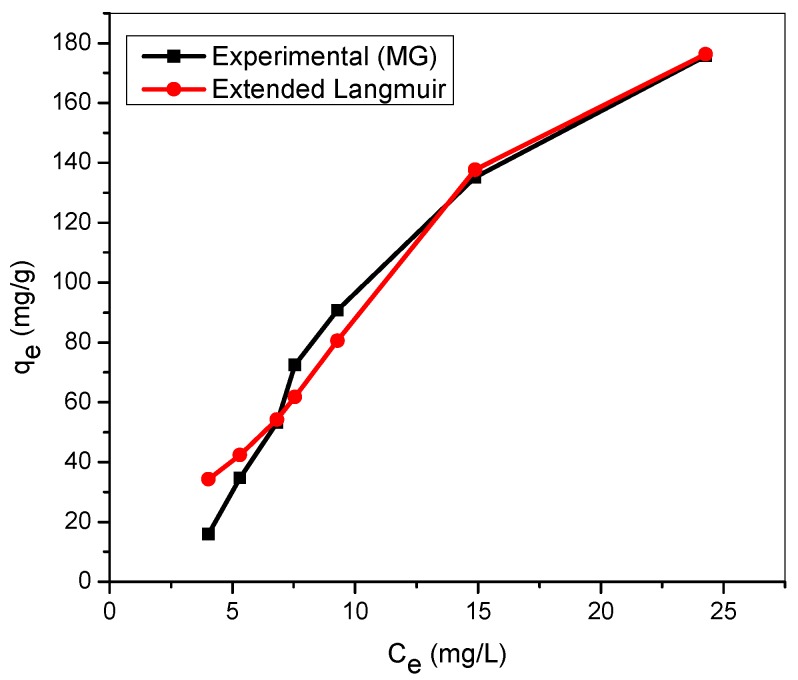
Extended Langmuir model for MG in binary system with MB (temperature: 25 °C; agitation speed: 100 rpm; dosage: 0.5 g/100 mL, Conc. MG = Conc. MB: (20–200 mg/L)).

**Figure 10:**
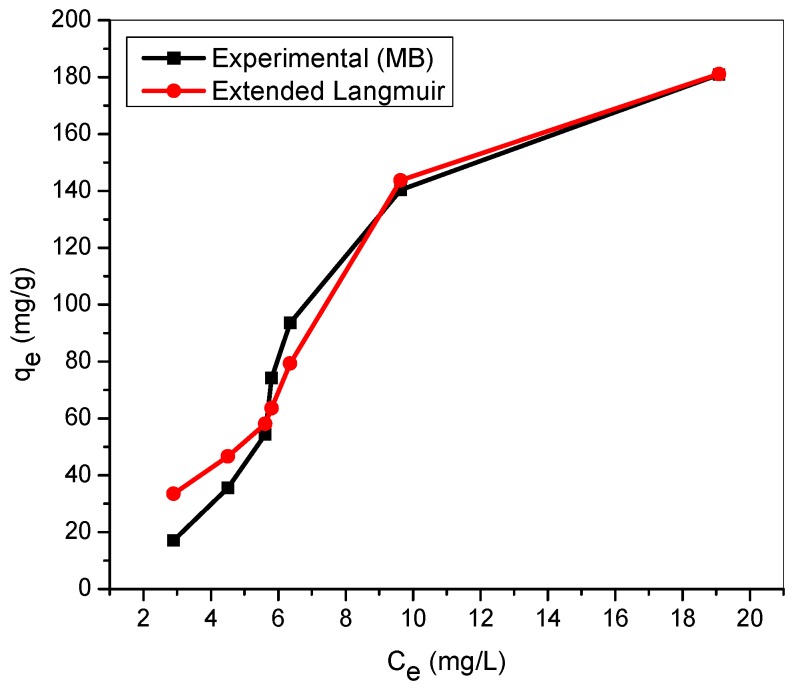
Extended Langmuir model for MB in binary system with MG (temperature: 25 °C; agitation speed: 100 rpm; dosage: 0.5 g/100 mL, Conc. MG = Conc. MB: (20–200 mg/L)).

Figure 9 and Figure 10 illustrated the assessment of experimental adsorption data with theoretical values generated from the extended Langmuir equations for binary cationic dye solution. The sum of squares error, SSE, estimated between the experimental adsorption capacity and extended Langmuir isotherm prediction for MG and MB in binary solution are 618 and 736, respectively (Table 4). The application of the extended Langmuir equations in the extrapolation of adsorption capacity of TMPAA for MG and MB dyes results in good agreement with the experimental adsorbent capacity especially at higher initial dye concentrations. However, the fundamental assumptions upon which the Langmuir model is based gave no lateral interaction between adsorbed moieties and equal independent rivalry between adsorbate components. This outcome is supported by the research findings of Yang and team (2016) for competitive adsorption of heavy metal ions using modified green tea waste [55].

#### 3.5.2. Extended Freundlich Equation (EFE) for Binary Cationic Dye System

In a binary cationic dye (MG and MB) mixture, the individual adsorption capacity ($q_{e,MG}$, $q_{e,MB}$) is estimated according to the following extended Freundlich models (derived from Equations (11) and (12)):

(16) qe,MG = 9.341 (Ce,MG) (11.179 ) + x1Ce,MGx1 + y1 Ce,MBz1

(17) qe,MB = 0.028 (Ce,MB) (11.283 ) + x2Ce,MBx2 + y2 Ce,MGz2

**Figure 11:**
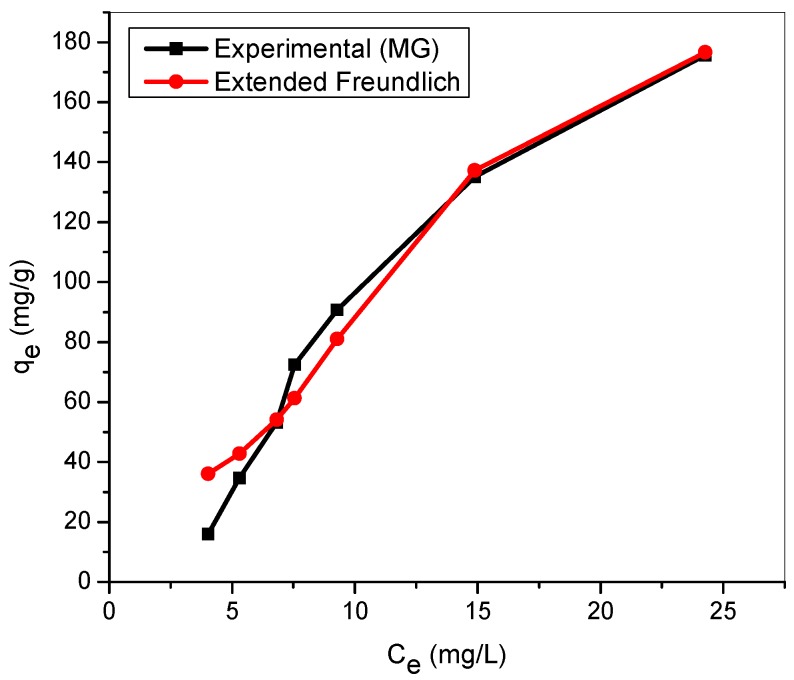
Extended Freundlich model for MG in binary system with MB (temperature: 25 °C; agitation speed: 100 rpm; dosage: 0.5 g/100 mL, Conc. MG = Conc. MB: (20–200 mg/L)).

**Figure 12:**
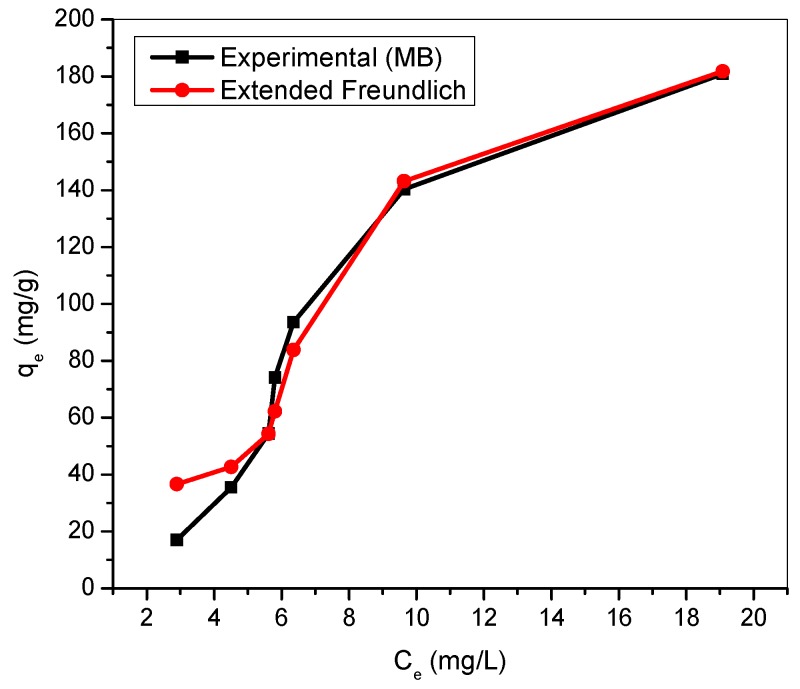
Extended Freundlich model for MB in binary system with MG (temperature: 25 °C; agitation speed: 100 rpm; dosage: 0.5 g/100 mL, Conc. MG = Conc. MB: (20–200 mg/L)). Table 6 displays the competition constants and the interactive effects of MG and MB in the binary system. The calculated P factor ($P_{fi}$) for both cationic dyes was greater than one, indicating that the adsorption of component i (MG/MB) is inhibited by the presence of another component (MB/MG) [37]. Besides, the higher value of the inhibitory effect (ΔIE) demonstrates that the entrapment of MG is suppressed in the presence of MB dye. This is corroborated by the estimated selectivity ratio, $S_{\left(MG/MB\right)}$ value that is greater than unity. The results confirmed that the TMPAA polymeric adsorbent has more affinity towards MG than MB [57]. Similar antagonistic analogous trends could be noticed also in the binary dyes solution of remazol brilliant blue (RBB) and disperse orange (DO) [58], methylene blue (MB) and methyl orange (MO) [59], and methyl orange (MO) and phenol [41].

Figure 11 and Figure 12 show the comparison of the experimental sorption capacity with the predicted values by extended Freundlich model for binary dye system. The isotherm was found suitable for the description of MG and MB adsorption, simultaneously. This phenomenon agreed with the findings of Remenarova et al. (2009) for the binary biosorption of malachite green (MG), auramine O (BY2), and thioflavine T (BY1) by moss *rhytidiadelphus squarrosus* [56]. The model parameters obtained and the sum of squares errors are tabulated in Table 5. The SSE values, 696 (MG), 681 (MB) are found comparable relatively to the one presented in the extended Langmuir isotherm (618 for MG; 735 for MB). However, both extended Langmuir and extended Freundlich failed to account for the type of interactions that existed between the cationic MG and MB molecules.

### 3.6. Adsorption Kinetics

**Figure 13:**
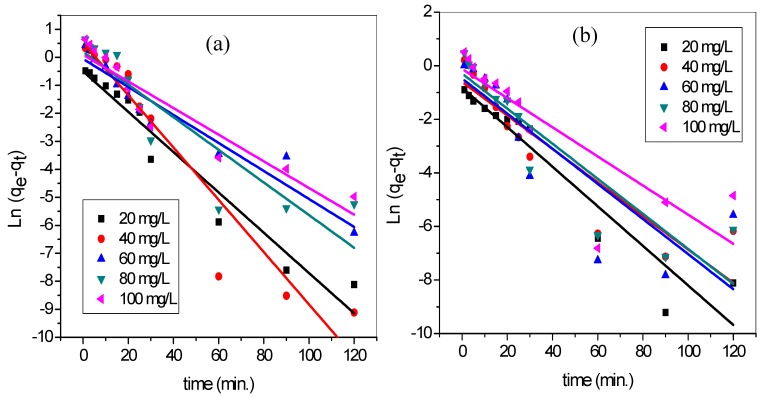
PFO model for (**a**) MG and (**b**) MB adsorption in binary system with MB (temperature: 25 °C; speed: 100 rpm; dose: 0.5 g/100 mL; Conc. MG = Conc. MB).

**Figure 14:**
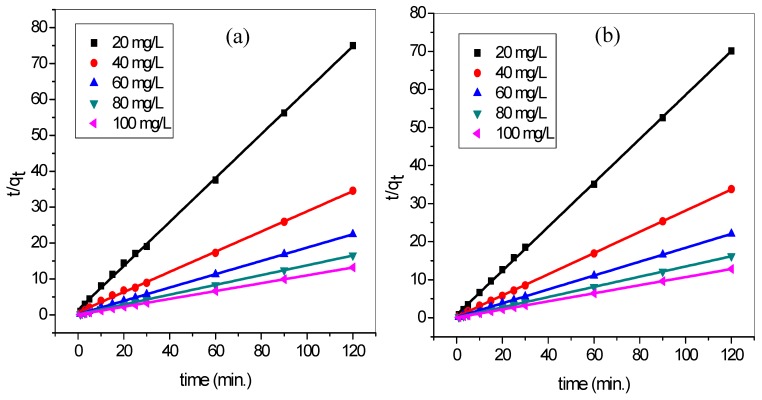
PSO model for (**a**) MG and (**b**) MB adsorption in binary system with MB (temperature: 25 °C; speed: 100 rpm; dose: 0.5 g/100 mL; Conc. MG = Conc. MB).

In order to determine the order and kinetics of the binary adsorption process, pseudo-first order (PFO) and pseudo-second-order (PSO) models were tested. The kinetic data were fitted into the PFO (Figure 13) and PSO (Figure 14) for the adsorption of MG and MB dye by TMPAA in a binary system. The kinetic factors and correlation coefficient gotten are presented in Table 7.

The PFO equation did not rank as favorable based on the correlation coefficient $R^{2}$ values (0.7035–0.9471). The MG and MB dye equilibrium concentration on the TMPAA, $q_{e(cal)}$ did not correlate to the experimental values $q_{e(\exp)}$, this further discrediting its suitability. Conversely, the PSO kinetic model adjudged well fitted the adsorption data with higher $R^{2}$ values (0.999–1). In addition, the calculated values of $q_{e(cal)}$ showed good agreement with the corresponding experimental values $q_{e(\exp)}$ for the range of initial concentrations investigated. This confirmed the suitability of the PSO model for the description of the adsorption process. Thus, the sorption process involves transferring and or sharing of electron between anionic TMPAA adsorbent and cationic dyes. Therefore, chemisorption was found to be the rate-limiting step, controlling the adsorption of MG and MB molecules onto TMPAA.

### 3.7. FTIR and SEM Analyses

**Figure 15:**
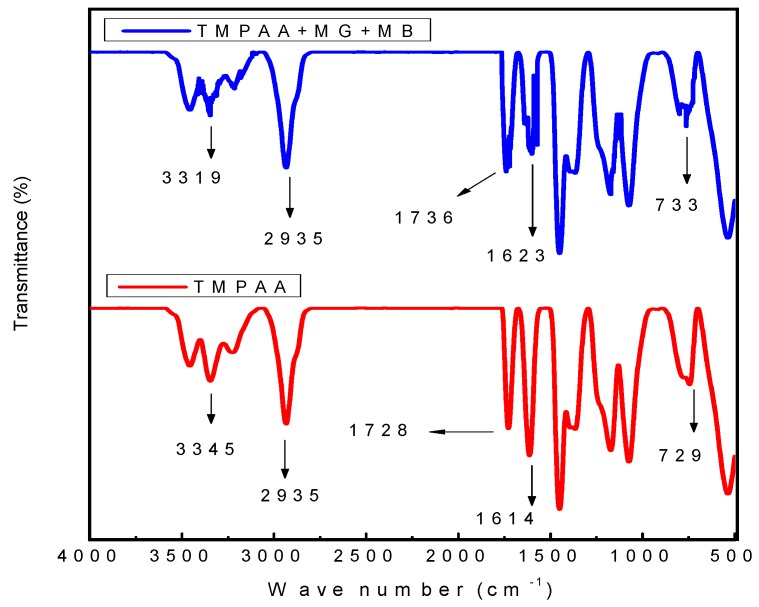
FTIR spectra of synthesized TMPAA (red) and TMPAA loaded with MG and MB dyes (blue).

Fourier transform infrared (FTIR) spectroscopy and scanning electron microscopy (SEM) are potent tools for delineating adsorption mechanism. FTIR spectra were recorded for the TMPAA adsorbent before and after dye uptake in the 4000–500 cm^−1^ (Figure 15). Some absorption peaks of the polymeric adsorbent are found slightly shifted in the spectrum of the MG and MB dye loaded adsorbent. The peak at 3345 cm^−1^ (-NH_2_/-OH), 1728 cm^−1^ (-C=O), and 729 cm^−1^ (-C=S) in unloaded adsorbent are shifted to 3319 cm^−1^, 1736 cm^−1^, and 733 cm^−1^ on the TMPAA after coadsorption of MG and MB cationic dyes. These characteristic surface-functional groups are essential to the sorption capacity of the adsorbent. Thus, strong interactions exist between adsorbate and adsorbent [54,60], probably consisting of inner-sphere surface complexation. This is due to electrostatic attraction between TMPAA-MG/MB. A similar phenomenon was reported by [61] on the adsorption of chromotrope dye onto activated carbon and [62] for the uptake of emerging pharmaceutical contaminants.

**Figure 16:**
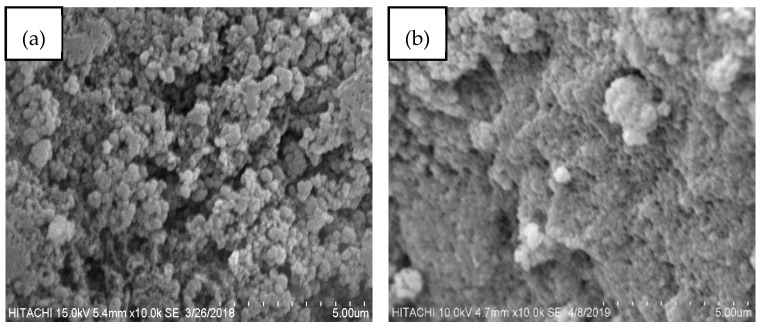
SEM micrographs of (**a**) TMPAA and (**b**) TMPAA loaded with MG and MB dyes.

Insightful information concerning the morphological features of the TMPAA is provided by SEM analysis. SEM image of the unloaded adsorbent (Figure 16a) shows the rough and non-uniform surface of the TMPAA. Post cationic dyes adsorption, a significant change is seen in the structure of the adsorbent (Figure 16b). Its voids appear to be occupied and covered with shinning and bulky molecules of the MG and MB dye. Lata et al. (2008), Hameed et al. (2017) and Sekulic group (2019) reported similar findings [61,62,63].

## 4. Conclusions

The competitive adsorption of cationic MG and MB dye under equimolar conditions on TMPAA adsorbent was investigated. The comparison delineates an antagonistic interaction between the MG and MB molecules, which mutually hindered the adsorption of both dyes on the TMPAA. The polymeric adsorbent shows higher affinity towards MG entrapment than MB in the binary system, viz. 150 mg/g against 124 mg/g at equilibrium. Extended Langmuir and extended Freundlich equations give a good estimation of the binary equilibrium data. Pseudo-second-order model fits suitably the coadsorption kinetics, which indicates chemisorption between the dye ions and TMPAA sorbent. According to this study, TMPAA could prove to be an attractive functional adsorbent when discrimination in the separation of cationic dyes from liquid phase is essential. This work could also offer theoretical guidance for the treatment of multicomponent industrial wastewater.

## Figures and Tables

**Table 1:** General properties of cationic MG and MB dyes.

Name of the Commercial Dye	Malachite Green, MG	Methylene Blue, MB
Colour Index Name	Basic Green 4	Basic Blue 9
λ _max_ (nm)	617	665
Molecular Weight (g/mol)	364.92	319.85
Charge	(+)	(+)
Chemical Formula	C_23_H_25_ClN_2_	C_16_H_18_ClN_3_S

**Table 2:** Adsorption isotherms constants for adsorption of MG and MB dye in single system.

Dye	Langmuir		Freundlich	
MG	$q_{\max}$ (mg/g)	429.18	$K_{F}$ (L/mg)	9.341
	$K_{L}$ (mg/g)	0.0188	n	1.179
	$R^{2}$	0.8137	$R^{2}$	0.9990
MB	$q_{\max}$ (mg/g)	308.64	$K_{F}$ (L/mg)	0.028
	$K_{L}$ (mg/g)	0.0476	n	1.283
	$R^{2}$	0.8927	$R^{2}$	0.9994

**Table 3:** Langmuir isotherm constants at 25 °C for the adsorption of MG and MB dyes on TMPAA in the binary system.

Dye in a Binary System	Langmuir Constants		
	$q_{\max}$(mg/g)	$K_{L}$(L/mg)	$R^{2}$
MG	150.97	0.0021	0.8357
MB	124.61	0.0017	0.9309

**Table 4:** Parameters of the extended Langmuir model for the binary adsorption of MG and MB by the TMPAA.

Dye in a Binary System	Model Constants		
	$K_{1}$	$K_{2}$	SSE
MG	−0.0629	0.0891	618.12
MB	−0.1281	0.1734	735.52

**Table 5:** Parameters of the extended Freundlich model for the binary adsorption of MG and MB by the TMPAA.

The Dye in the Binary System	Model Constants			
	$x_{i}$	$y_{i}$	$z_{i}$	SSE
MG MB	−3.412	−0.095	−3.095	695.90
	−0.1281	0.1734	735.52	680.66

**Table 6:** Competition constants and interaction effects of MG and MB by the TMPAA in a binary solution.

The Dye in a Binary System	Competition Constants			
	$P_{fi}$	ΔIE	$S_{\left(i/j\right)}$	Interactive Effect
MG	2.843	0.648	1.2115	Antagonistic
MB	2.477	0.596	0.8254	Antagonistic

**Table 7:** Kinetic parameters and correlation coefficient for PFO and PSO kinetic models for adsorption MG and MB by TMPAA in the binary system.

The Dye in a Binary System	Initial Dye Concentration $C_{o}$ (mg/L)	$q_{e(\exp)}$ (mg/g)	PFO Kinetic Model			PSO Kinetic Model		
			$q_{e(cal)}$ (mg/g)	$k_{1}$ (min^−1^)	$R^{2}$	$q_{e(cal)}$ (mg/g)	$k_{2}$ (g/mg·min)	$R^{2}$
MG	20	1.60	0.60	0.072	0.9471	1.63	0.268	0.9993
	40	3.47	1.67	0.093	0.9128	3.56	0.108	0.9989
	60	5.35	0.95	0.050	0.9209	5.39	0.181	0.9993
	80	7.25	1.21	0.058	0.8092	7.34	0.107	0.9996
	100	9.10	1.10	0.048	0.8843	9.16	0.139	0.9999
MB	20	1.71	0.44	0.074	0.9048	1.73	0.433	0.9997
	40	3.55	0.55	0.063	0.8221	3.58	0.285	0.9999
	60	5.44	0.62	0.066	0.7082	5.48	0.214	0.9999
	80	7.42	0.75	0.065	0.8101	7.46	0.225	1
	100	9.37	0.88	0.054	0.7035	9.42	0.169	1
